# Microstructure and Strength of Yttria-Reinforced Aluminum by ARB

**DOI:** 10.3390/ma19143015

**Published:** 2026-07-13

**Authors:** Amirhossein Meysami, Farshad Rahimi, Alex Meisami, Sayyed Erfan Aghili, Mohammad Meysami

**Affiliations:** 1 Chemical & Biomolecular Engineering Department, Clarkson University, Potsdam, NY 13676, USA; ameysami@clarkson.edu; 2 Materials Engineering Group, Golpayegan College of Engineering, Isfahan University of Technology, Golpayegan 87717-67498, Iran; gimail.fa@gmail.com (F.R.); aghili@iut.ac.ir (S.E.A.); 3 Judd Leighton Schools of Business and Economics, Indiana University South Bend, South Bend, IN 46634, USA; ameisami@iu.edu; 4 Department of Mathematics, The University of Tulsa, Tulsa, OK 74104, USA

**Keywords:** accumulative roll bonding (ARB), aluminum matrix nanocomposites, Y_2_O_3_ nanoparticles, severe plastic deformation, mechanical properties

## Abstract

The effects of accumulative roll bonding (ARB) on the microstructure and mechanical properties of an AA 1060/Y_2_O_3_ nanocomposite are investigated. AA 1060 alloy sheets that had been pre-rolled to a thickness of 1 mm were annealed at 430 °C, stacked with Y_2_O_3_ nanoparticles, and roll-bonded with a 50% thickness reduction per pass up to five passes. FESEM images, tensile testing (ASTM E8), and Vickers hardness measurements were used to characterize the material. With an increasing number of ARB passes, the dispersion of the Y_2_O_3_ particles within the AA 1060 sheets improved, as indicated by the increasing value of the dispersion index *D* (from 0.35 at pass 1 to 0.81 at pass 5); the ultimate tensile strength of the nanocomposite sheets increased from 77.5 MPa for the as-annealed sheets to 187 MPa for the sheets after five passes of roll bonding, and the hardness of the sheets reached 57.5 HV after five passes of roll bonding (≈2.4× the hardness of the annealed sheets). Furthermore, the elongation of the nanocomposite sheets decreased sharply after the first pass of roll bonding but then stabilized between 3.5 and 5%. A physics-guided saturation model is fitted to the measured UTS data, and leave-one-out cross-validation is used to assess its limited predictive capability.

## 1. Introduction

In the past few years, there has been growing interest in the development of metal products with grain sizes smaller than one micron in diameter. These types of metals are referred to as ultrafine-grained (UFG) metals, and they exhibit not just an increase in strength compared to metals that have larger grains but also increased formability [1,2,3]. Furthermore, nanostructured metals have proven to be highly successful within a variety of consumer markets, from the production of defense and aerospace equipment [4] to the production of transportation and medical equipment [5]. For instance, nanostructured metals are now being used in the production of prostheses and implants [6], as well as in the manufacture of drug delivery systems [7]. Furthermore, nanostructured metals are also being utilized in the manufacture of components for airplanes, passenger cars, and even internal combustion engines, and the use of these metals in these industries is having a significant impact upon the aviation industry itself [8,9].

The production of ultrafine-grained materials is mainly based on two things. The first is that the application of a high plastic strain leads to the production of a high dislocation density inside the material. Furthermore, the shift and reorganization of these dislocations as the manufacturing process continues leads to the formation of a grain-boundary network. This method allows UFG materials to achieve better mechanical properties while preserving their original chemical composition [10].

Severe plastic deformation (SPD) is an important part of metal forming. SPD involves putting a great deal of strain into a solid material without observing any clear changes in the dimensions of that solid. SPD processes can be repeatedly applied to metals to allow for the accumulation of strain until the grains within the material become refined [11,12]. Processes like rolling and extrusion are common SPD processes but have limited potential to introduce high amounts of strain into metals. Conventional deformation processes are used to create large-sized materials through plastic deformation. Due to this, researchers are paying closer attention to SPD processes that can apply a great deal of strain to metals at low temperatures while maintaining the same cross-sectional area of the material. Such developments aim to work around the limitations of conventional metal forming processes.

The advantages of employing such methods are related to their reliance on high hydrostatic pressure, which improves the metal’s plasticity and enables stronger strains to be applied without causing cracks within the sample. Moreover, despite the small amount of initial strain that typically occurs during the application of severe plastic deformation (SPD) methods, repeated application leads to the imposition of a severe cumulative strain on the sample [13,14]. In 1998, the application of a rolling-based plastic deformation technique was publicized. This process, known as accumulative roll bonding (ARB) [15], is notable for its ability to continuously produce superfine materials and, thus far, remains the only severe plastic deformation process that relies on rolling as the sole means of changing the shape of the processed material [16,17,18]. The various methods have been combined in research studies to provide a complete overall understanding of the properties of carbon materials and their structural stability [19].

In addition to these applications, aluminum metal and its alloys are employed in numerous other fields, including electronic technology, automotive structures, marine applications, renewable energy management, and more. The use of aluminum in these applications results from its many benefits, including high specific strength, lightweight properties, good formability, notable resistance to corrosion, enhanced conductivity, environmentally friendly characteristics, and recyclability, among others [20,21,22,23,24]. Despite the excellent properties of Al metal, it has low tensile strength. Hence, to enhance its strength for various high-stress applications, aluminum requires strengthening, which can be achieved through processes such as precipitation hardening [25,26] or solid-solution strengthening [27,28].

Another approach that can be followed to enhance the strength of aluminum alloys is the development of aluminum composites [29]. Composite materials are formed through the combination of two or more different materials that have distinct and separable physical and chemical properties while retaining their individual identities within the final composite. As a result, composites often exhibit characteristics that cannot be obtained from any single constituent alone. Metal matrix composites (MMCs) are a type of composite material in which physical, mechanical, and electrical properties are enhanced by incorporating strong reinforcements into a soft matrix metal. Such composites generally utilize metals such as aluminum, titanium, magnesium, and copper as base materials [30]. Recently, researchers have successfully developed aluminum matrix composites with reinforcing particles, known as aluminum metal matrix composites (AMMCs) [31].

The most commonly used types of reinforcements in AMMCs include SiC, Si_3_N_4_, TiC, and Al_2_O_3_ [32]. Rajesh et al. carried out a study on the wear behavior of Al7075 alloy using different weight fractions of SiC and Al_2_O_3_ [33]. The results showed that the composite’s wear resistance improved with the increased weight fraction of the reinforcements. Similarly, Shalaby et al. produced A359 alloy composites with different weight percentages of SiC and Si_3_N_4_ particles [34]. Upon analyzing the microstructure, the researchers noted a uniform and consistent distribution of hybrid reinforcements throughout the composite matrix. Additionally, Prasad et al. explored the production of aluminum 7075 matrix composites with varying weight proportions of TiC [35]. The study found that increasing the quantity of TiC particles within the aluminum matrix improved mechanical attributes such as ultimate tensile strength and hardness, at the expense of reduced ductility.

Yttrium oxide (Y_2_O_3_, or yttria), an oxide ceramic known for its refractoriness with a melting point of 2430 °C, exhibits exceptional thermal stability exceeding 1000 °C. Its notable attributes include high strength, hardness, and thermal conductivity [36]. Adding yttria to aluminum as an AMMC reinforcement can noticeably improve strength, corrosion resistance, and wear resistance [37,38,39]. Yttrium oxide also shows low reactivity with aluminum in both the solid and liquid phases, which makes it a suitable reinforcement for aluminum.

From a cost standpoint, the use of Y_2_O_3_ as an addition to Al alloys is also worthy of consideration. Commercial-grade Y_2_O_3_ powder of 99.5% purity costs approximately $20–50 per kilogram, a cost higher than those of both Al_2_O_3_(∼$1–3/kg) and SiC (∼$3–8/kg). However, there are a few factors that justify the use of Y_2_O_3_ powder and make it an attractive candidate for inclusion in aluminum alloys for targeted high-performance applications: the relatively low amount of Y_2_O_3_ that is required to provide the desired enhancement to the aluminum alloy (only 2 wt.% in this study); its low reactivity with the aluminum matrix in both solid and liquid states, which helps avoid interfacial degradation products that can form with SiC or Al_2_O_3_ at elevated temperatures; and the thermal stability of the Y_2_O_3_ particles, which is of interest for applications in the aerospace, defense, and biomedical industries, where long-term performance at elevated temperature outweighs raw-material costs.

The preparation of the AMMC Al–Y_2_O_3_ composites was carried out using the accumulative roll bonding (ARB) process. Y_2_O_3_ nanoparticles were dispersed during the first cycle, followed by successive cumulative roll bonding for up to five cycles.

Despite the promising properties of Y_2_O_3_ as a potential type of reinforcement for aluminum matrix composites, most of the studies that have used Al/Y_2_O_3_ composites have used sintering or powder metallurgy processes to produce the composites [37,38,39]; a systematic investigation combining ARB processing, quantitative microstructural characterization using a defined dispersion index, and a data-driven analysis fitted to experimental measurements is absent from the literature. Thus, the main objectives of the current investigation are: (i) to fabricate Al/Y_2_O_3_ nanocomposites via the ARB process, using up to five passes, and to characterize the microstructure of the nanocomposites through image analysis; (ii) to investigate the relation between the dispersion index of the nanocomposites and their mechanical properties; and (iii) to evaluate the applicability of a physics-guided, data-driven model, fitted directly to the experimental data, to describe the strengthening of the nanocomposites and assess its predictive capability.

## 2. Materials and Methods

### 2.1. Materials

**Figure 1:**
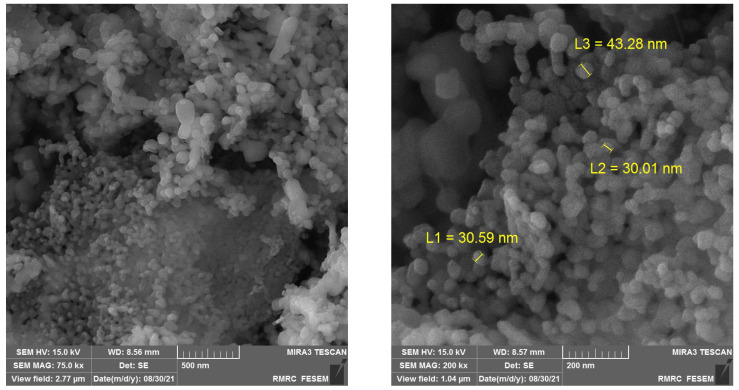
FESEM images of primary yttria nanoparticles used as the reinforcement for the Al alloy metal matrix.

A 1 mm thick AA 1060 aluminum sheet was used as the metallic matrix. To keep the material composition accurate, the alloy sheet was analyzed by a WAS Foundry Master model quantometer (Hünenberg, Switzerland), and the results are listed in Table 1. Sigma Aldrich (St. Louis, MO, USA) yttria nanoparticles were used as reinforcement for the Al alloy metal matrix. These yttria nanoparticles have an average particle size of 50 nm with a purity of 99.5%. Their particle morphology and average size are presented in FESEM images shown in Figure 1.

### 2.2. ARB Process on the Sheets

Initially, the sheets were cut into strips measuring 20 × 4 cm^2^, following a cutting direction parallel to the rolling orientation of the primary AA 1060 sheets. Subsequently, the 1 mm thick sheets were subjected to an annealing process at 430 °C for 20 min. to relieve residual stresses from prior cold rolling; this annealing treatment did not alter the sheet thickness. Two aluminum sheets were stacked, and Y_2_O_3_ nanoparticles corresponding to 2 wt.% of the final composite mass were weighed and sprayed uniformly between them using an atomizer. They were then secured using copper wire to prevent slippage during the rolling process. It is important to note that the time between brushing and feeding the sheet into the rolling machine should be kept under 5 min. to avoid forming aluminum oxide. The rolling was carried out on a rolling machine with a 50% reduction in thickness.

For each ARB pass with a 50% thickness reduction, the true strain in the thickness direction is given by $\varepsilon =\ln(t_{0}/t_{1})$, where $t_{0}$ and $t_{1}$ are the sheet thicknesses before and after a single pass, respectively. The corresponding von Mises equivalent strain under the commonly used plane-strain rolling assumption is denoted by $\varepsilon_{eq}$, and the accumulated equivalent strain after *N* passes is denoted by $\varepsilon_{\mathrm{total}}$ [40]:

(1) $$\begin{array}{cc} \varepsilon & =\ln\left(\frac{t_{0}}{t_{1}}\right)=\ln\left(2\right), \end{array}$$

(2) $$\begin{array}{cc} \varepsilon_{eq} & =\frac{2}{\sqrt{3}}\ln\left(2\right), \end{array}$$

(3) $$\begin{array}{cc} \varepsilon_{total} & =N\;\varepsilon_{eq}. \end{array}$$

**Figure 2:**
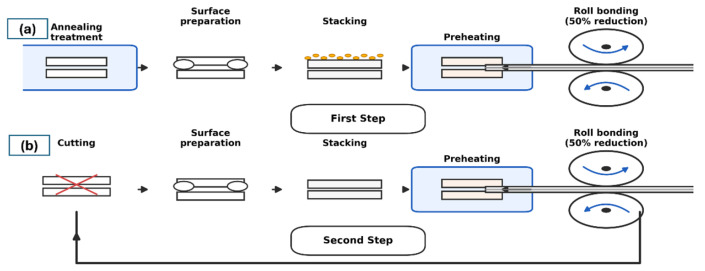
Schematic illustration of the fabrication steps of the Al/Y_2_O_3_ composite by accumulative roll bonding: (**a**) first ARB step, including annealing, surface preparation, stacking with Y_2_O_3_ nanoparticles, preheating, and roll bonding with a 50% thickness reduction; and (**b**) subsequent ARB steps, including cutting, surface preparation, restacking, preheating, and repeated roll bonding with a 50% thickness reduction.

The total equivalent strain increases linearly with the number of ARB passes. The machine employed in this research is a 35-ton rolling machine (2-roller type) with rollers measuring 145 mm in diameter. To establish the connection between the sheets, the rotation speed of its rollers was set at 6 rpm. The bonded samples were cut into two, degreased, and brushed. The samples were stacked again and then rolled further, with the goal of getting another 50% reduction in thickness. Subsequently, the rolling procedure was repeated for an additional four passes. Prior to each rolling pass, the sheet underwent a 30 s preheating at a temperature of 150 °C. Figure 2 shows a schematic illustration depicting the accumulative roll bonding process for an AA 1060 aluminum sheet.

**Figure 3:**
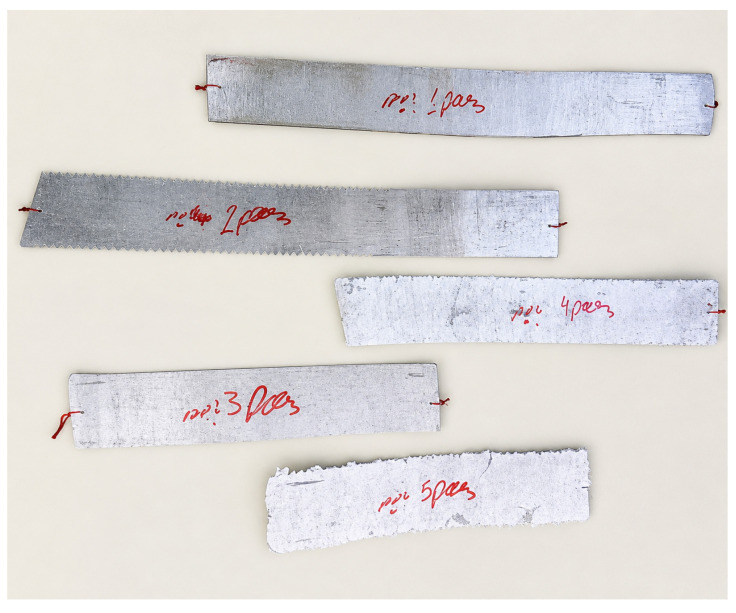
Aluminum reinforced with yttria nanoparticles (Al/Y_2_O_3_) after five ARB passes.

The distribution of yttria (Y_2_O_3_) nanoparticles in the obtained composite and the sheets’ adhesion were examined in a field-emission scanning electron microscope (FESEM). Figure 3 shows the Al/Y_2_O_3_ composite sheets after five ARB passes.

**Figure 4:**
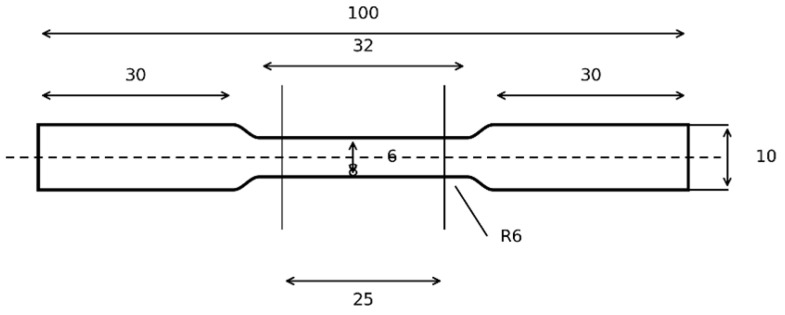
Schematic of the tensile test specimen according to ASTM E8 (dimensions in mm).

Tensile test specimens were prepared from the sheets processed at different rolling cycles according to ASTM E8 [21]. The gauge width and gauge length were considered to be 6 mm and 25 mm, respectively (Figure 4). Tensile loading was applied parallel to the rolling direction. Specimens were extracted from the central region of each rolled sheet (avoiding free-edge zones) so that the gauge length was parallel to the rolling direction (RD), which minimizes the influence of strain inhomogeneity across the sheet width. Uniaxial tensile tests were performed using a Hounsfield H50K (Hounsfield Test Equipment Ltd., Redhill, UK) testing machine at a crosshead speed of 0.25 mm/min, corresponding to a strain rate of $1.67\times 10^{-4}$ $s^{-1}$, at room temperature. The strength and elongation of the specimens were obtained. Tensile results are reported as the average of three specimens for each composite [1]. Since composite properties can differ in different directions, tensile tests in this study were conducted only along the sheet rolling direction (RD).

Hardness measurements were performed on specimens prepared from the cross-section of the rolled sheets (RD–ND plane). After surface preparation, Vickers hardness was measured at 10 randomly selected points, and the average value is reported. A load of 3 kg was applied for 15 s.

## 3. Experimental Results and Discussion

### 3.1. Microstructural Evolution

**Figure 5:**
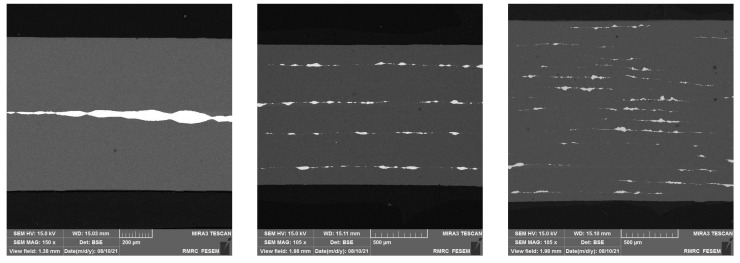
FESEM micrographs of the Al/Y_2_O_3_ composite after the first, third, and fifth ARB passes: **left**, first pass; **middle**, third pass; and **right**, fifth pass.

Figure 5 presents the microstructure of the Al/Y_2_O_3_ composite after the first, third, and fifth passes, as observed by FESEM. As shown in Figure 5 (left), after the first pass the powder layer is sharp and distinct, with a clear delineation between the two Al sheets. During rolling, after each pass, the sheets undergo plastic deformation and their length increases [23,24]. With increasing ARB cycles, the powder layer progressively breaks up, and fragmentation occurs.

**Figure 6:**
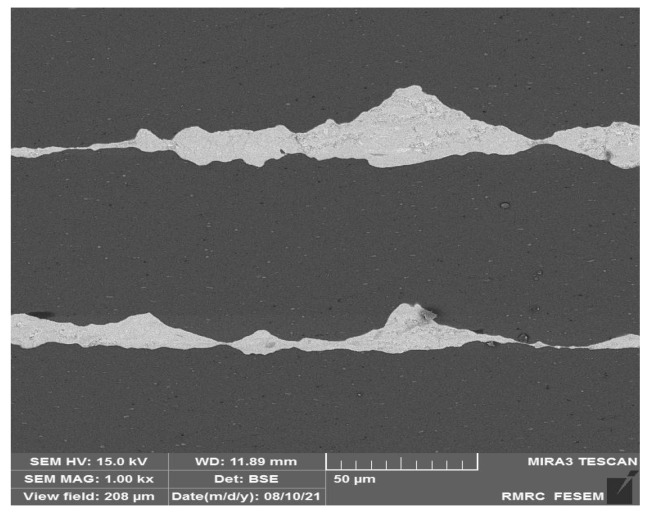
FESEM image showing remaining particle agglomerates and/or very thin particle layers in the Al/Y_2_O_3_ composite after ARB.

In some regions, large agglomerates of particles are observed; however, in most regions the particles are distributed within the aluminum matrix and become more uniformly dispersed (Figure 5 middle) [24,25]. After the fifth pass, the particles are mostly dispersed in the aluminum matrix (particle-free regions are reduced); nevertheless, large particle clusters and very thin powder layers can still be observed (Figure 5 (right) and Figure 6).

Reinforcing particles in composites can hinder dislocation motion and increase the dislocation density through the Orowan mechanism [12], thereby refining the matrix microstructure and leading to grain refinement [24,26].

The quality of the bonding between the Al sheets changes with the number of ARB passes, which impacts the effective strength of the resulting composite material. After the first pass (Figure 5 left), the powder layer is still sharp, and there is still a clear interface between the two Al sheets, indicating that the sheets have not been fully bonded together; this region of incomplete bonding can act as a stress concentrator and is thought to contribute to the sharp drop in the ductility of the material following pass 1 [34]. Although the 30 s preheating of the samples to 150 °C prior to each pass helps to disrupt the oxide layer on the Al sheet surfaces and promote metallic bonding [15], each pass of 50% reduction in thickness at near-room temperature is generally insufficient to bond the two sheets completely [17]. With increasing numbers of passes, however, the interface between the two sheets becomes less distinct (Figure 5 middle and right), indicating improved bonding between the sheets after five passes. Because direct grain-boundary imaging was not performed, the grain sizes reported in Table 2 should be interpreted as approximate values estimated from Hall–Petch-type analysis and supported by literature values for ARB-processed commercial-purity Al under similar processing conditions [17].

### 3.2. Quantitative Microstructural Analysis

In addition to the qualitative FESEM observations in Figure 5 and Figure 6, a quantitative image-based analysis was performed to obtain measurable microstructural parameters.

For each ARB condition (one, three, and five passes), three representative FESEM micrographs were acquired at identical magnification. Image analysis was performed using ImageJ software, version 1.54g. Y_2_O_3_ particles were segmented from the aluminum matrix using a grayscale thresholding procedure (Otsu automatic threshold method). The analyzed area in each micrograph was on the order of 50×50μ$m^{2}$, and the reported values correspond to the average of three micrographs.

The following parameters were evaluated: (i) estimated particle-layer thickness (where continuous layers were present), (ii) agglomerate size range (largest connected particle clusters), (iii) estimated interparticle spacing λ, (iv) dispersion index *D* based on cluster area fraction. The dispersion index was defined as

(4) $$D=1-\frac{A_{c}}{A_{t}},$$

where $A_{c}$ is the projected area of particle clusters larger than 2 μm, and $A_{t}$ is the total analyzed area. Higher *D* values correspond to improved particle dispersion. The quantitative results are summarized in Table 3.

Using the calculated values, the dispersion level can be defined quantitatively as: Low (D<0.4), Moderate (0.4≤D≤0.7), and High (D>0.7). Thus, the one-pass condition (D=0.35) is classified as Low, the three-pass condition (D=0.62) as Moderate, and the five-pass condition (D=0.81) as High. This quantitative linkage helps minimize subjectivity and associates the dispersion level with a measurable cluster area fraction.

The progressive reduction in particle-layer thickness, agglomerate size, and interparticle spacing with increasing number of passes indicates that repeated severe plastic deformation during ARB promotes particle fragmentation and redistribution within the aluminum matrix, leading to the observed strengthening.

### 3.3. Mechanical Properties

#### 3.3.1. Tensile Test

**Figure 7:**
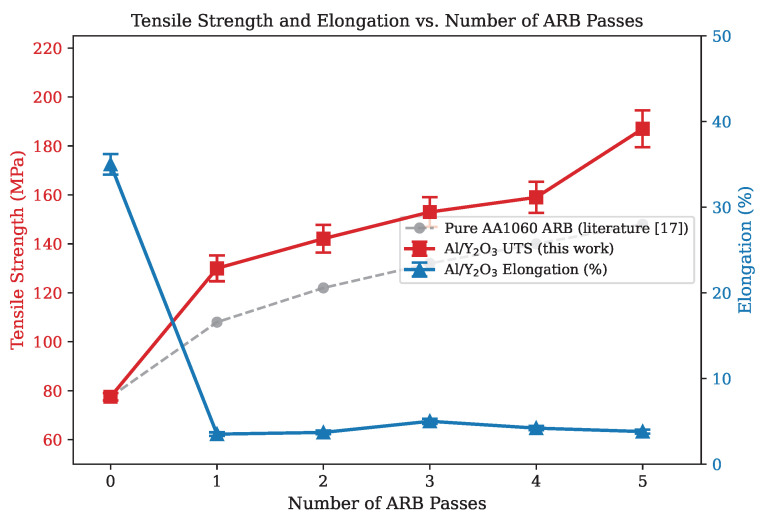
Variation in tensile strength and elongation versus the number of ARB passes for the Al/Y_2_O_3_ composite (this work) and for ARB-processed pure AA1060 without reinforcement (literature [17]). Error bars represent ±1 standard deviation from three replicate specimens. The composite reaches 187 MPa after five passes, surpassing the 145–165 MPa typically reported for pure AA1060 under similar ARB conditions [17].

Figure 7 shows the tensile strength and elongation of the Al/Y_2_O_3_ composite produced by ARB, together with literature data for ARB-processed pure AA1060 without reinforcement [17], as a function of the number of ARB passes; error bars represent one standard deviation from three replicate specimens. The strength rose dramatically after the first cycle compared to the annealed condition, increasing from 77.5 MPa (annealed) to 130 MPa. The maximum ultimate tensile strength (UTS) of the nanocomposite was obtained after five passes, reaching 187 MPa.

The percentage improvement in mechanical properties was calculated using

(5) $$\%\mathrm{Increase}=\frac{P_{N}-P_{0}}{P_{0}}\times 100,$$

where *P* denotes a mechanical property (e.g., UTS or hardness), $P_{0}$ is the value in the annealed condition, and $P_{N}$ is the value after *N* ARB passes.

In the initial cycles, the distribution of reinforcing particles in the composite is not uniform, and they are observed as a layer or as agglomerates; several cycles are required for the particles to become sufficiently dispersed in the aluminum matrix (Figure 5). This effect becomes more pronounced in the final pass and leads to a significant improvement in strength compared with the annealed sample.

The UTS of ARB-processed pure AA1060 without any reinforcement is around 145–165 MPa after five passes under similar conditions (gray dashed line in Figure 7) [17]. The Al/Y_2_O_3_ composite reaches around 187 MPa after the same number of passes, which is an improvement of around 14–28% due to the addition of Y_2_O_3_ particles through Orowan looping, thermal-mismatch-induced dislocations, and particle-assisted grain refinement.

It should be noted that the dispersion of reinforcing particles in the metal matrix has a strong influence on the composite properties. Uniform particle distribution improves certain mechanical properties; however, according to some studies, non-uniform dispersion not only fails to improve mechanical properties but may also reduce them in some directions [11,27]. It has also been reported that a minimum number of passes is necessary so that particle dispersion becomes suitable and the porosity present in the sample is reduced [28].

As shown in Figure 7, the tensile strength increases rapidly in the first pass(es), mainly due to work hardening. Increasing the dislocation density and the formation of micro-scale shear bands raise the strength of the material [11,29]. In the second and third passes, the rate of strength increase decreases as the contribution of work hardening becomes less dominant, and grain refinement based on the Hall–Petch relationship is cited as the main reason for further strengthening [11].

In the following relations, $\sigma_{y}$ is the yield strength, $\sigma_{0}$ is the baseline yield strength of the matrix, *d* is the average grain size, and $k_{HP}$ is the Hall–Petch constant. Particle-related strengthening is described using $\Delta \sigma_{Orowan}$ (Orowan looping) and $\Delta \sigma_{\rho }$ (dislocation-density/Taylor strengthening), where *G* is the shear modulus, *b* is the Burgers vector, λ is the interparticle spacing, *r* is the particle radius, α is a constant of order unity, and ρ is the dislocation density [41].

The grain-refinement contribution to strengthening can be expressed using the Hall–Petch relationship:

(6) $$\sigma_{y}=\sigma_{0}+k_{HP}\;d^{-1/2}.$$

In nanoparticle-reinforced composites, two additional mechanisms can also contribute to strengthening. The first is the Orowan mechanism: For reinforcement particles smaller than 1 μm, dislocations bypass the particles and form dislocation loops around them. As the uniformity of particle distribution improves with increasing ARB passes, the effective interparticle spacing decreases, thereby increasing the Orowan contribution to strengthening [11].

The Orowan looping contribution can be written as

(7) $$\Delta \sigma_{Orowan}=\frac{0.13Gb}{\lambda }\ln\;\left(\frac{r}{b}\right).$$

The contribution from dislocation-density strengthening (Taylor relation) is approximated by

(8) $$\Delta \sigma_{\rho }=\alpha Gb\sqrt{\rho }.$$

Accordingly, the overall yield strength can be summarized conceptually as

(9) $$\sigma_{y}\approx \sigma_{0}+\Delta \sigma_{HP}+\Delta \sigma_{Orowan}+\Delta \sigma_{\rho }.$$

Two sources of temperature differential within the process contribute to the thermal mismatch stresses that develop within the composite material. First, each pass of the rolling operation is preceded by a 30 s preheating of the sheet stack at 150 °C. The large difference in the coefficients of thermal expansion (CTEs) of aluminum (24 ×$10^{-6}$ $K^{-1}$) and yttria (8.1 × $10^{-6}$ $K^{-1}$) [30,31] leads to the development of thermal mismatch stresses at the particle–matrix interface after heating. Second, the rolling operation is performed rapidly enough that adiabatic conditions partially apply during portions of the rolling operation, so local plastic dissipation transiently raises the temperature in the deformation zone. Both effects promote the generation of geometrically necessary dislocations around the Y_2_O_3_ nanoparticles within the sheet [11,20], contributing to the observed strengthening.

Regarding elongation, it is worth noting that ductility drops sharply in the first rolling pass. The elongation decreases from 35% in the annealed sample to 3.5% in the Al/Y_2_O_3_ composite. This reduction is mainly due to severe work hardening introduced in the first pass, which increases the dislocation density and lowers ductility [33]. In addition, insufficient bonding between the two sheets and the presence of interconnected layers can act as stress concentrators, which has been reported as another contributing factor [34,35].

The significant drop in ductility at pass 1 (from 35% to 3.5%) is consistent with a brittle mode of fracture for the composites. The brittle fracture mode is likely due to the fact that the interface between the two Al sheets is not well bonded, and that the initial planar particle layer acts as a preferential crack initiation site under the tensile loading that is applied to the specimens. Beyond pass 2, however, the elongation stabilizes to between 3.5% and 5%. The reason for this recovery in ductility is likely due to each of the following factors: (i) the progressive elimination of the sharp interface (Figure 5 middle and right), (ii) the grain-refined matrix that is formed during the ARB process and impedes crack propagation, and (iii) improved bonding quality, which reduces stress concentration [33,34]. These same types of fracture mode transitions have been reported for other aluminum composites processed via the ARB process [17].

Starting from the second pass, under the application of higher strain, the elongation does not decrease but rather tends towards a certain level and might even increase. The reasoning behind the improvement in elongation from the second pass onward is somewhat similar to that which is true of the tensile strength; the higher strain causes a more refined microstructure than that of the previous pass, leading to a microstructure that diminishes the negative effect of dislocation accumulation. Moreover, the refined structure hinders crack propagation, which further contributes to improved elongation [33,34].

#### 3.3.2. Hardness Test

**Figure 8:**
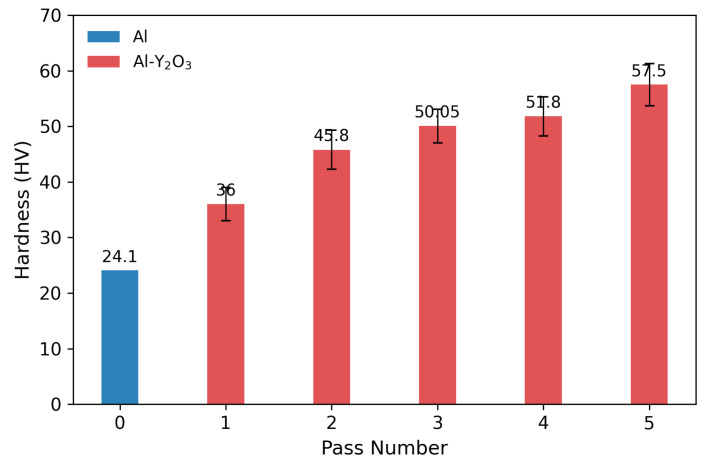
Variation in Vickers hardness of annealed aluminum and the Al/Y_2_O_3_ composite produced by ARB.

Figure 8 shows the variation in hardness of the Al/Y_2_O_3_ composite produced by ARB. The hardness of annealed aluminum is also included for comparison.

For the Al/Y_2_O_3_ composite, the hardness after the first rolling pass is 36 HV, indicating a 50% increase compared with that of the annealed sample. With increasing rolling passes, the hardness further increases. This increase is sometimes small and sometimes more pronounced. In the last cycle, the hardness reaches its maximum value of 57.5 HV, corresponding to a 138% increase compared with the annealed condition (i.e., about 2.4 times).

The variation in hardness with ARB passes reflects the mechanisms that contribute to the strengthening, as explained in Section 3.3.1. For instance, in the early passes, work hardening dominates, and the high dislocation density introduced by the 50% rolling reduction accounts for the majority of the pass 1 hardness increase. Additionally, the grain size is reduced with increasing passes of ARB, from ∼45 μm for the annealed condition to ∼0.9 μm for pass 5 (Table 2), indicating an increase in the contribution of Hall–Petch strengthening to the alloy strength ($\Delta \sigma_{HP}\approx 59$ MPa in Section 4). Furthermore, the interparticle spacing (λ) is also reduced with increasing passes of ARB, from 3–6 μm for pass 1 to 1–3 μm for pass 5 (Table 3), indicating a modest increase in Orowan strengthening. The dispersion index of the particles within the alloy is also increased from 0.35 for pass 1 to 0.81 for pass 5. Thus, the hardness increase with increasing number of ARB passes reflects the combined effects of work hardening, grain refinement, Orowan strengthening, and reduced porosity.

Accordingly, the hardness evolution is consistent with the observed tensile-strength trends.

In the initial passes, the reinforcing particles are not uniformly distributed in the matrix. With each rolling pass, particle dispersion in the matrix improves and the porosity decreases; therefore, the hardness continues to increase up to five passes. In general, the factors affecting hardness during accumulative roll bonding include: surface brushing before rolling, work hardening due to friction between the rolls and the sheet, grain refinement, the presence of reinforcement particles between the sheets, reduction in porosity in the sheets [24], fragmentation of the aluminum oxide layer during rolling, and dispersion of these oxide particles into the matrix [11].

## 4. Data-Driven Process–Property Analysis

The analysis of the Al/Y_2_O_3_ ARB composite presented in this section relies entirely on the data measured in Section 3. The analysis is based upon the following: a physics-guided saturation model that predicts the ultimate tensile strength (UTS) of the composite and was fitted by nonlinear least squares to the measured UTS values, a quantitative decomposition of the contribution of each strengthening mechanism to the strength of the composite using the microstructural data from Table 2 and Table 3, a formal correlation between the measured dispersion index of the composite and its strength, and an analysis of the predictive capability of the model, as determined by leave-one-out cross-validation (LOO-CV).

### 4.1. Physics-Guided Saturation Model Fitted to Experimental Data

The trend of UTS with the pass number of ARB exhibits a saturation-like trend, which is indicative of the progressive exhaustion of both work-hardening capacity and grain-refinement driving force.

A physics-guided saturation model [11] was fitted directly to the five experimental UTS measurements from the ARB passes:

(10) $$\sigma \left(N\right)=\sigma_{sat}-\left(\sigma_{sat}-\sigma_{ann}\right)\;e^{-kN},$$

where $\sigma_{ann}=77.5$ MPa is the fixed value of the ultimate tensile strength (UTS) of the annealed material, $\sigma_{sat}$ is the fitted saturation strength, and *k* is the fitted rate parameter. Nonlinear least-squares fitting provides a saturation strength of $\sigma_{sat}=184$ MPa and a saturation rate of k=0.49. The coefficient of determination ($R^{2}$) for this fit is $R^{2}=0.77$, and the root mean squared error (RMSE) is RMSE =9.2 MPa.

**Figure 9:**
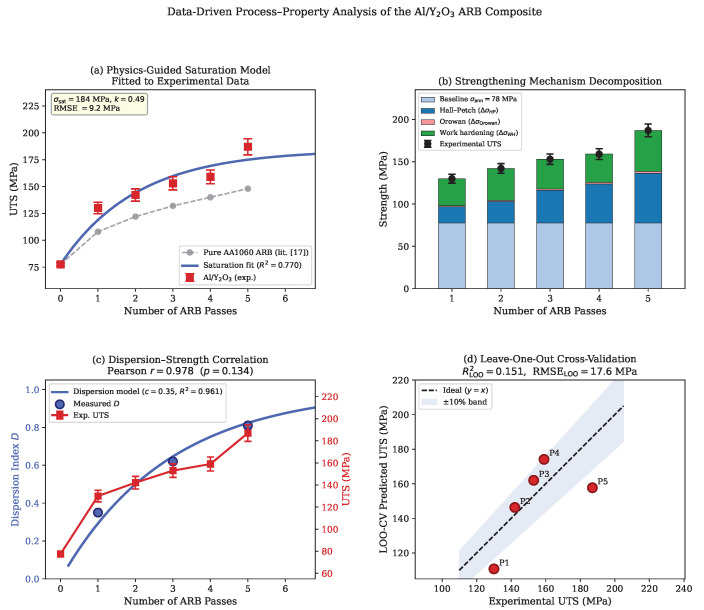
Data-driven process–property analysis of the Al/Y_2_O_3_ ARB composite. (**a**) The physics-guided model for saturation of strength in Al alloys (Equation (10)), obtained by fitting the model to the experimental UTS data from passes 1 to 5 of the ARB process ($\sigma_{ann}=77.5$ MPa is fixed at the value for annealed Al), is plotted alongside the literature curve for pure Al and all data points from the experiments with error bars; the fitted parameters are $\sigma_{sat}=184$ MPa and k=0.49 ($R^{2}=0.77$, RMSE =9.2 MPa). (**b**) The contributions to the strength increase from the strengthening mechanisms, including the baseline ($\sigma_{ann}$), Hall–Petch increment ($\Delta \sigma_{HP}$), Orowan increment ($\Delta \sigma_{Orowan}$), and work-hardening residual ($\Delta \sigma_{WH}$), are plotted together with the experimental UTS data; the grain sizes and interparticle spacing values used in the calculation are presented in Table 2 and Table 3. (**c**) The model for the dispersion of Y_2_O_3_ particles in Al ($\eta \left(N\right)=1-e^{-cN}$) was fitted to the three measured dispersion indices (c=0.35, $R^{2}=0.96$); the experimental data for ultimate tensile strength (UTS) is also plotted on the right axis. The Pearson correlation between *D* and UTS from passes 1, 3, and 5 is r=0.978. (**d**) The cross-validation parity plot of the saturation model; the large residual for the data point at pass 5 (P5) reflects the strength jump associated with the rapid increase in the dispersion index between passes 3 and 5 (*D* changed from 0.62 to 0.81).

Figure 9a displays the fitted model along with the experimental data and the literature curve for pure-Al ARB. The model slightly underpredicts the UTS at pass 5 (∼187 MPa), with the difference between the model-predicted and experimental values being approximately 12 MPa. Between passes 3 and 5, the dispersion index increases from 0.62 to 0.81, and the mean interparticle spacing decreases from 2–4 to 1–3 μm (Table 3). Thus, the enhanced Orowan contribution and increased rate of grain refinement in this regime are not fully described by the single-exponential model. Hence, the use of descriptors related to the microstructure beyond pass number alone may allow for more accurate modeling of UTS.

### 4.2. Strengthening Mechanism Decomposition

To provide an approximate interpretation of the strengthening mechanisms, the experimental UTS at each pass is expressed as

(11) $$\mathrm{UTS}\left(N\right)=\sigma_{ann}+\Delta \sigma_{HP}\left(N\right)+\Delta \sigma_{Orowan}\left(N\right)+\Delta \sigma_{WH}\left(N\right),$$

where $\sigma_{ann}=77.5$ MPa is the baseline, $\Delta \sigma_{HP}$ is the incremental Hall–Petch contribution relative to the annealed state, $\Delta \sigma_{Orowan}$ is the Orowan contribution, and $\Delta \sigma_{WH}$ is the work-hardening residual.

The Hall–Petch increment uses the grain sizes from Table 2:

(12) $$\Delta \sigma_{HP}\left(N\right)=k_{HP}\;\left[d{\left(N\right)}^{-1/2}-d_{0}^{-1/2}\right],$$

with $k_{HP}=65$ MPa·μ$m^{1/2}$ for Al [41]. The Orowan increment uses the interpolated λ values from Table 3, *G* = 26,500 MPa, $b=2.86\times 10^{-4}\;\mu$m, and r=0.025μm:

(13) $$\Delta \sigma_{Orowan}\left(N\right)=\frac{0.13Gb}{\lambda (N)}\ln\;\left(\frac{r}{b}\right).$$

The work-hardening residual $\Delta \sigma_{WH}=\mathrm{UTS}-\sigma_{ann}-\Delta \sigma_{HP}-\Delta \sigma_{Orowan}$ represents the remaining strength contribution after the estimated Hall–Petch and Orowan terms are subtracted; it includes the combined effects of dislocation accumulation, porosity reduction, bonding improvement, and uncertainty in the estimated grain sizes.

As depicted in Figure 9b, the Hall–Petch contribution increases from around 19 MPa in pass 1 to around 59 MPa in pass 5 as the grain size decreases from ∼5 μm to ∼0.9 μm. These estimates suggest that grain refinement provides an important strengthening contribution in the later passes of the deformation process. Furthermore, the Orowan contribution is relatively small, varying between 1 and 2 MPa, reflecting the interparticle distances measured via FESEM imaging (1–6 μm) being relatively large compared to the Burgers vector length. Finally, the work-hardening residual contribution (≈33–48 MPa) is relatively constant across passes, likely due to the saturation of dislocations with increasing deformation.

### 4.3. Dispersion–Strength Correlation

The measured dispersion index *D* (Table 3) is also associated with the UTS through two different analyses. The first involves fitting the exponential dispersion model

(14) $$\eta \left(N\right)=1-e^{-cN},$$

to the three different measured values of *D* at passes 1, 3, and 5. The fit to these three data points reveals that c=0.35 with $R^{2}=0.96$ (Figure 9c). This type of relationship is expected for systems that approach a steady-state dispersion limit with increasing deformation, which is consistent with the progressive breakdown of agglomerates observed by FESEM.

The second analysis involves determining the Pearson correlation coefficient for the three pairs of measured (D,UTS) values. The coefficient is found to be r=0.978, which indicates a strong positive correlation between dispersion and UTS. While the small sample size (n=3) suggests caution in drawing conclusions from this coefficient, the *p*-value of p=0.13 indicates that more data is needed to statistically confirm the existence of such a relationship. However, the observed relationship is in accord with the expected relationship between improved dispersion, Orowan strengthening, and grain refinement.

### 4.4. Leave-One-out Cross-Validation

Given the small size of the available data set, leave-one-out cross-validation was performed to assess the predictive capability of the saturation model (Equation (10)). The model was refitted using four passes of the experiment and used to predict the withheld pass. As shown in Figure 9d, the predictions for passes 1–4 are within ±10% of the experimental results for those passes; however, the predicted value for pass 5 is substantially below the measured value of 187 MPa (${\mathrm{RMSE}}_{\mathrm{LOO}}=17.6$ MPa, $R_{\mathrm{LOO}}^{2}=0.15$).

The relatively poor performance of the model in pass 5 indicates that the jump in UTS between pass 4 and pass 5 (+28 MPa), which coincided with an increase in the dispersion index from 0.62 to 0.81, could not be accurately extrapolated from the more gradual trend observed in the early passes of the experiment. The implication of this result is twofold: the saturation model should only be used to interpolate within the available data set rather than to extrapolate beyond it; an alternative microstructure-aware model that considers parameters like the dispersion index *D* or interparticle spacing λ should be developed to improve the accuracy of the predictions. Building such a model would require a larger database of experimental results, spanning multiple Y_2_O_3_ weight fractions, ARB temperatures, and reduction rates, which will be pursued in future work.

## 5. Conclusions

An AA1060/Y_2_O_3_ nanocomposite was fabricated using the accumulative roll bonding (ARB) process for up to five passes and characterized using FESEM, tensile testing, and Vickers hardness measurements. The index *D* of particle dispersion increased monotonically from 0.35 for pass 1 (Low) to 0.62 for pass 3 (Moderate) and to 0.81 for pass 5 (High), confirming progressive particle redistribution with accumulated deformation. The ultimate tensile strength of the nanocomposite increased from 77.5 MPa for the annealed material to 187 MPa after five passes of the ARB process (an increase of 141%), which is 14–28% higher than that of the AA1060 alloy processed using the same technique but without Y_2_O_3_ reinforcement (≈145–165 MPa). The Vickers hardness of the nanocomposite increased from 24.1 HV for the annealed material to 57.5 HV after five passes of the ARB process (≈2.4×), following the tensile-strength trend and reflecting the combined effects of work hardening, grain refinement, and particle-assisted strengthening. The elongation of the material dropped from 35% for the annealed material to 3.5% for pass 1, consistent with brittle fracture promoted by intensive work hardening and incomplete bonding, and thereafter remained within the range of 3.5% to 5% for passes 2 to 5 as bonding quality improved and the refined microstructure inhibited crack propagation.

An approximate decomposition of the strength of the nanocomposite into Hall–Petch, Orowan, and residual work-hardening contributions was used to interpret the measured ultimate tensile strength (UTS) values. Based on the approximate grain-size estimates, the Hall–Petch contribution after five ARB passes is estimated to be ≈59 MPa for d≈0.9μm, while the Orowan contribution is estimated to be much smaller, ≈2 MPa, for interparticle spacings of 1–3 μm. A saturation model directly fitted to the measured UTS data gives $\sigma_{sat}=184$ MPa and k=0.49 ($R^{2}=0.77$, RMSE =9.2 MPa), but this model should be interpreted mainly as a descriptive fit within the available experimental range. Leave-one-out cross-validation of the saturation model indicates a limited generalization capability ($R_{\mathrm{LOO}}^{2}=0.15$) for the model; the significant jump in the UTS of the nanocomposite after five passes of the ARB process cannot be predicted from the slowly increasing UTS values for passes 1 to 4. Thus, future work should focus upon creating a microstructure-aware model that incorporates parameters like *D* or λ as the model’s input variables.

## Figures and Tables

**Table 1:** Chemical composition of AA 1060 aluminum alloy (D = 2.705 g/cc, tensile strength = 77.5 MPa).

Element	Al	Si	Fe	Cu	Mn	Zn	Ti	V	Pb	Co
%wt.	99.6	0.12	0.21	0.003	0.01	0.03	0.005	0.017	0.005	0.01

**Table 2:** Estimated grain size and Vickers hardness of the Al/Y_2_O_3_ composite under different ARB conditions. Grain sizes are estimated from Hall–Petch analysis of the experimental tensile data and corroborated by literature values for ARB-processed commercial-purity Al under similar conditions [17]; direct FESEM grain-boundary imaging was not performed in this study.

ARB Condition	Estimated Grain Size (μm)	Vickers Hardness (HV)
Annealed (0 passes)	∼45	24.1
1 pass	∼5	36.0
3 passes	∼1.8	50.1
5 passes	∼0.9	57.5

**Table 3:** Quantitative microstructural descriptors of the Al/Y_2_O_3_ composite at different ARB passes based on FESEM image analysis.

Pass	Layer Thickness (μm)	Agglomerate Size (μm)	λ (μm)	*D*
1	8–12	5–15	3–6	0.35
3	2–5	2–8	2–4	0.62
5	<1	1–4	1–3	0.81

## Data Availability

The original contributions presented in this study are included in the article. Further inquiries can be directed to the corresponding author.
